# Supplementary material for: Ionotropic Receptor-dependent moist and dry cells control hygrosensation in Drosophila
Source: eLife. 2017 Jun 16;6:e26654. doi: 10.7554/eLife.26654 (PMC5495567; doi:10.7554/eLife.26654)
Supplement: Source code 1. — DOI: http://dx.doi.org/10.7554/eLife.26654.010 [file elife-26654-code1.docx]

function FunImaging_analysis_Garrity()

% Original analysis script written by Timothy Wiggin (Leslie Griffith lab)

% Edited by Brian Cary for Garrity Lab

%%%%%%%%%%%%%%%%%%%%% ASSUMPTIONS %%%%%%%%%%%%%%%%%%%%%%.

% 16-bit TIFF file inputs %

%%%%%%%%%%%%%%%%%%%%%%%%%%%%%%%%%%%%%%%%%%%%%%%%%%%%%%%%

%% Set analysis parameters

CODE_REVISION_DATE = '2017-2-28'; % Code version stamp for output

ALIGNMENT_TOL = 1; %Can mess with if alignment is weird. Lower is

stricter.

%%%%%%%%

% Change based on experimentn

% Are used to calculate maximum change from baseline fit

BASELINE_FRAMES = 20;

PRE_BASELINE_LENGTH = 28;

STIMULUS_LENGTH = 40;

%%%%%%%%

% Set data read paths

[~,path]=uigetfile('*.tif','Select folder of tif files');

file_names = dir(fullfile(path, '*.tif'));

% Set data save folder

data_save_dir = uigetdir(pwd,'Select folder to save data output');

% Prompt for annotation

expt_title = inputdlg('Provide a brief title for the experiment:',

'Annotation Prompt');

% Prompt for alignment or not

alignyesno = inputdlg('Perform alignment? [y/n]');

%% Read in image data

fprintf(1,'Reading image data:(%03d%%)', 0); % Progress bar

first_frame = imread(fullfile(path, file_names(1).name));

frame_series = zeros(size(first_frame, 1), size(first_frame, 2),

length(file_names));

frame_series(:,:,1) = first_frame;

for i = 2:length(file_names)

fprintf(1,'\b\b\b\b\b%03d%%)', round( (i / length(file_names))

* 100)); % Progress bar

frame_series(:,:,i) = imread(fullfile(path,

file_names(i).name));

end

fprintf(1,'\n');

%% Correct Drift in the Imagesn

if strcmp(alignyesno, 'y');

disp('Correcting for drift...');

aligned_images = align_stacks(frame_series, ALIGNMENT_TOL);

else

aligned_images = frame_series;

end

%% Select Signal and Background ROIs

disp('Calculating signal intensity and ratio...');

doodle_image = mean(aligned_images, 3);

figure;

imshow(doodle_image);

imshow(doodle_image/max(doodle_image(:)));

title('Draw polygon surrounding: Analysis region Of interest');

signal_mask = roipoly();

close;

figure;

imshow(doodle_image/max(doodle_image(:)));

title('Draw polygon surrounding: Background Region');

background_mask = roipoly();

pause(0.1)

close all;

pause(0.2)

%% Calculate signal to bg Ratio

signal_mask = double(signal_mask);

signal_mask_size = sum(signal_mask(:));

background_mask = double(background_mask);

background_mask_size = sum(background_mask(:));

signal = zeros(size(aligned_images,3),1);

background = zeros(size(aligned_images,3),1);

masked = zeros(size(aligned_images));

for i = 1:size(aligned_images,3)

masked(:,:,i) = aligned_images(:,:,i) .* signal_mask;

signal(i) = sum(sum(masked(:,:,i))) / signal_mask_size;

temp = aligned_images(:,:,i) .* background_mask;

background(i) = sum(temp(:)) / background_mask_size;

end

corrected = signal - background;

scaled_signal = corrected / mean(corrected(1:BASELINE_FRAMES));

output = struct('Analysis_Code_Version', CODE_REVISION_DATE, ...

'Brief_Experiment_Title', expt_title, ...

'Reference_Image', doodle_image, ...

'ROI_Mask', signal_mask, ...

'BG_Mask', background_mask, ...

'Channel_ROI_Intensity', signal, ...

'Channel_BG_Intensity', background, ...

'Scaled_Signal', scaled_signal);

% Display a figure of output

figure

subplot(3,1,1)

hold on

plot(output.Channel_BG_Intensity, 'color', 'cyan')

ylabel('Background Intensity')

subplot(3,1,2)

hold on

plot(output.Channel_ROI_Intensity, 'color', 'magenta')

ylabel('ROI Intensity')

subplot(3,1,3)

plot(output.Scaled_Signal, 'color', 'green')

ylabel('Normalized Signal/Background')

%%%%

% Fits a line to baseline data and finds the maximum difference

between

% linear fit and signal

try

y = output.Scaled_Signal;

y = y(~isnan(y));

x = (1:length(y)).';

P = polyfit(x(1:PRE_BASELINE_LENGTH),y(1:PRE_BASELINE_LENGTH),

1);

yfit = P(1)*x +P(2);

drug_time = PRE_BASELINE_LENGTH:(PRE_BASELINE_LENGTH +

STIMULUS_LENGTH);

max_change = max(abs(yfit(drug_time)-y(drug_time)));

fprintf('The max change in signal from baseline is %.3f \n',

max_change)

catch

disp('baseline or stimulus length does not fit. No max change

calculated.');

end

%%%%

%% Write the data to disk

%fprintf(1,'Writing output to disk:(%03d%%)', 0); % Progress bar

out_fdl = strcat('Analysis_', expt_title, '_', datestr(now, 29) );

mkdir(data_save_dir,out_fdl{1});

save(fullfile(data_save_dir, out_fdl{1}, 'analysis_output.mat'),

'output');

%imwrite(uint16(frame_series(:,:,1)), fullfile(pwd, out_fdl{1},

'original_frames.tif'));

% Write a csv with the roi,bg, and signal data

fid = fopen(fullfile(data_save_dir, out_fdl{1}, [out_fdl{1},

'.csv']), 'w');

fprintf(fid, '%s,', 'ROI');

fprintf(fid, '%s,', 'BG');

fprintf(fid, '%s\n', 'Scaled Signal');

fclose(fid);

csvdata(:,1) = output.Channel_ROI_Intensity;

csvdata(:,2) = output.Channel_BG_Intensity;

csvdata(:,3) = output.Scaled_Signal;

dlmwrite(fullfile(data_save_dir, out_fdl{1}, [out_fdl{1}, '.csv']),

csvdata, '-append');

% Save alignment video if alignment was performed

if strcmp(alignyesno, 'y')

imwrite(uint16(aligned_images(:,:,1)), fullfile(data_save_dir,

out_fdl{1}, 'aligned_image.tif'));

%imwrite(uint16(masked(:,:,1)), fullfile(pwd, out_fdl{1},

'masked.tif'));

for i = 2:size(aligned_images, 3)

fprintf(1,'\b\b\b\b\b%03d%%)', round( (i /

size(aligned_images, 3)) * 100)); % Progress bar

%imwrite(uint16(frame_series(:,:,i)), fullfile(pwd,

out_fdl{1}, 'original_frames.tif'), 'WriteMode' , 'append');

%imwrite(uint16(aligned_images(:,:,i)), fullfile(pwd,

out_fdl{1}, 'aligned_image.tif'), 'WriteMode' , 'append');

end

end

fprintf('\n');

disp('Done!');

end

%%%%% alignment function. Recursively matches frames.

function [aligned_images] = align_stacks(images_to_align,

ALIGNMENT_TOL)

[optimizer, metric] = imregconfig('multimodal');

Rfixed = imref2d(size(images_to_align));

half_stack = round(size(images_to_align,3) / 2);

[aligned_images] = align_half(images_to_align(:,:,1:half_stack),

images_to_align(:,:,half_stack + 1:end));

function [a_images] = align_half(image_A, image_B)

% Check if the A stack is aligned to itself

tform = imregtform(image_A(:,:,end) , image_A(:,:,1) ,

'translation' , optimizer , metric);

if(pdist2([0 0], tform.T(3,1:2)) > ALIGNMENT_TOL)

% If there is only one frame in the stack, it is

already aligned to itself and this will never execute

ha_st = round(size(image_A,3) / 2);

[image_A] = align_half(image_A(:,:,1:ha_st),

image_A(:,:,ha_st + 1: end));

end

% Check if the B stack is aligned to itself

tform = imregtform(image_B(:,:,end) , image_B(:,:,1) ,

'translation' , optimizer , metric);

if(pdist2([0 0], tform.T(3,1:2)) > ALIGNMENT_TOL)

% If there is only one frame in the stack, it is

already aligned to itself and this will never execute

ha_st = round(size(image_B,3) / 2);

[image_B] = align_half(image_B(:,:,1:ha_st),

image_B(:,:,ha_st + 1: end));

end

% The A and B stacks are now internally aligned. Check if

the A

% and B stacks are aligned with one another

tform = imregtform(image_B(:,:,1) , image_A(:,:,1) ,

'translation' , optimizer , metric);

if(pdist2([0 0], tform.T(3,1:2)) > ALIGNMENT_TOL)

% Not aligned, apply the transformation to the entire B

stack

for i = 1:size(image_B,3)

image_B(:,:,i) = imwarp(image_B(:,:,i), tform,

'OutputView', Rfixed);

end

end

% The A and B stacks are now internally aligned and aligned

with

% one another. Concatonate and return them.

a_images = cat(3, image_A, image_B);

end

end
